# *Blakealtica*, a new genus of flea beetles (Coleoptera, Chrysomelidae, Galerucinae, Alticini) from the Dominican Republic

**DOI:** 10.3897/zookeys.959.53415

**Published:** 2020-08-14

**Authors:** Keezhpattillam Viswajyothi, Alexander S. Konstantinov

**Affiliations:** 1 Monte L. Bean Life Science Museum, Brigham Young University, Provo, Utah, U.S.A. 84602 and Kerala Agricultural University, Department of Agricultural Entomology, College of Agriculture, Vellayani, Trivandrum, Kerala, India Brigham Young University Provo United States of America; 2 Systematic Entomology Laboratory, USDA, c/o Smithsonian Institution, P. O. Box 37012, National Museum of Natural History, Washington, DC 20013-7012, USA National Museum of Natural History Washington United States of America

**Keywords:** *
Monomacra
*, Neotropical region, new taxa, sclerotized vagina, vaginal palpi, West Indies

## Abstract

*Blakealtica fusca* a new genus and new species from the Dominican Republic is described and illustrated. In addition to external features, beetle thoracic sclerites (including metendosternite) are fully examined and illustrated. *Blakealtica* is similar to *Monomacra* Chevrolat,1836 and related genera (*Disonycha* Chevrolat, 1836, *Hemilactica* Blake, 1937, *Myrmeconycha* Konstantinov & Tishechkin, 2017, *Parchicola* Bechyne & Bechyne, 1975, *Pseudodisonycha* Blake, 1954, and *Rosalactica* Bechyne & Bechyne, 1977) as all studied representatives of these genera are missing the sclerotized vaginal palpi or have them membranous and otherwise poorly developed. This feature may be unique for the *Monomacra* group of genera as it has not been seen anywhere else in flea beetles.

## Introduction

Flea beetles in the West Indies have received more attention than in other areas of Central and South America in recent years (Blake 1933–1954, Konstantinov 2002, Konstantinov and Konstantinova 2011, Micheli and Konstantinov 2019). However, to properly classify them in a coherent, morphologically sound, generic classification still requires establishments of new genera. Several new genera were recently created to classify flea beetles that inhabit moss cushions (e.g., Linzmeier and Konstantinov 2020). Some genera are proposed to accommodate taxa that were misclassified in the past [e.g., *Chaetocnema tuberculata* Suffrian, 1868 (Konstantinov and Linzmeier 2020)]. In this paper, a new genus, *Blakealtica*, is described for specimens that were collected in 1992 in the Dominican Republic by R. Woodruff and P. Skelley (FSCA) and also in 2014 by N. Woodley and the second author of this paper (USNM). All the specimens were collected at mid-altitude (ca. 400–500 meters in elevation), mostly at night with UV light traps, so the host plant of the species in question remains unknown.

## Materials and methods

Dissecting techniques and morphological terminology follow Konstantinov (1998). In addition, terminology for adult thoracic structures and ridges follows Lawrence and Ślipinski (2013), Lingafelter and Konstantinov (2000), and McHugh et al. (1997). Specimen labels are cited verbatim, according to the format justified previously (Konstantinov 1998, Konstantinov and Lingafelter 2002, and Konstantinov et al. 2011). Specimen observations were made with a Zeiss Stemi SV11 Apo microscope. Digital photographs of morphological structures were taken with Axio Zoom V16 microscope and AxioCam HRC digital camera attached to it and with AxioCam HRC Zeiss attached to Leitz Diaplan compound microscope. The specimens are deposited in collections of the National Museum of Natural History, Smithsonian Institution, Washington DC, USA (**USNM**); Florida State Collection of Arthropods, Tallahassee, FL, USA (**FSCA**); and Museo Nacional de Historia Natural, Santo Domingo, Dominican Republic (**MHND**).

## Taxonomy

### Blakealtica gen. nov.

Taxon classificationAnimaliaColeopteraChrysomelidae

3EC78878-C5B2-53D4-A9D3-936207CF142B

http://zoobank.org/B26C00CA-E531-43F3-B97F-E6CE21ED4C54

Figures 1 , 2–5 , 6–8 , 9, 10 , 11–16 , 17–20 , 21–24

#### Description.

Body oblong, narrow, flat in lateral view, length 4.16–5.51 mm, width 1.72–2.27; thickness 1.18–1.51. General color dark yellowish to brown with light metallic, blueish, or pinkish tint. Elytra and pronotum same color (Fig. 2).

**Figure 1.**
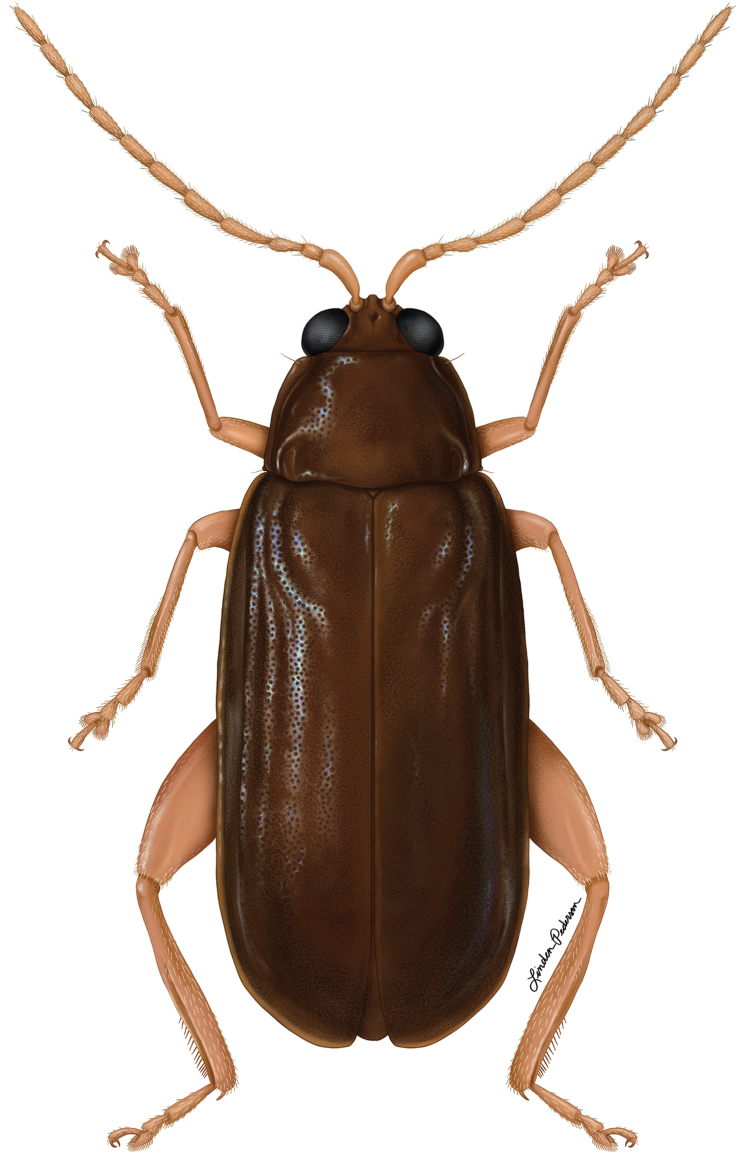
Adult *Blakealtica fusca*, illustration by Linden Pederson.

***Head*** (Figs 4, 5): Vertex sparsely and unevenly covered with closely placed round punctures, punctures without setae. Supraorbital pore well developed, easily noticed among other punctures. Midcranial suture present only in lower part, represented by short, relatively wide deep depression. Supraorbital, supracallinal, and orbital sulci absent. Midfrontal sulcus well developed, long, antennal calli and top of frontal ridge separated by distinct suprafrontal sulcus. Frontolateral sulcus absent. Antennal calli nearly trapezoidal or quadrate, as long as wide, not entering interantennal space. Surface of antennal callus same level as surface of vertex and surface of frontal ridge. Antennal calli shorter than frontal ridge. Antennal grooves between eye and frontal ridge present. Frontal ridge as wide anteriorly as posteriorly, continues straight between and below antennal sockets. Sides of frontal ridge parallel to each other. Posterior end of frontal ridge acute. Frontal ridge extends slightly between antennal calli, separated from vertex by antennal calli, generally long (ca. 2.75 longer than longitudinal diameter of antennal socket). Frontal ridge in lateral view slightly convex. Anterofrontal ridge extremely low, as thin as width of frontal ridge and occupying a fraction of space below antennal socket. Dorsal surface of anterofrontal ridge on either side of frontal ridge uneven, with visible convexity. Frontal and anterofrontal ridges form nearly right angle with each other in frontal view. Orbit extremely narrow, much narrower than transverse diameter of antennal socket, inner margin of eye slightly sinuate, diverging towards mouth parts. Distance between eyes (above antennal sockets) in frontal view subequal to transverse diameter of eye. Sides of head converging ventrally below eyes. Genal space narrow, one-sixth the longitudinal diameter of the eye. Clypeus pale, band-like. Labrum 0.4 times as long as wide, anterior margin entire, three pairs of labral setae in regular row, arising from basal third of labrum, extending beyond anterior margin of labrum (Fig. 11). Maxillary palpi slender with four palpomeres, basal palpomere slender, shortest, second slender, same length as the third, third thick, apical one conical (Fig. 14). Labial palpi with three palpomeres, basal one shorter and slightly narrower than the middle one, third conical (Fig. 12); mandible with five teeth-like projections (Fig. 13).

**Figures 2–5.**
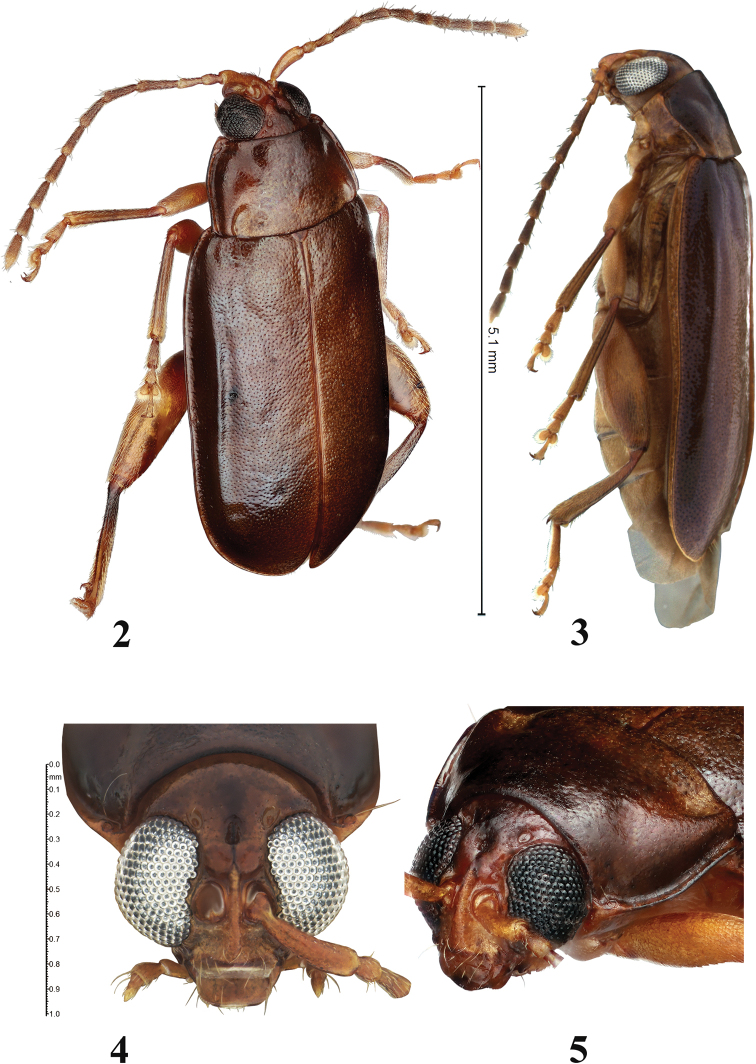
Adult *Blakealtica fusca*. **2** Habitus, three-quarter view **3** habitus, lateral view **4** head, frontal view **5** head three-quarter view.

***Antennae*** (Figs 2, 3): Antenna with eleven antennomeres, filiform, extending in females up to mid-elytron, in males beyond mid-elytron. Antennomere I, long, club-shaped, longer than II and III combined; II shorter than III, longer than half of III; II narrower than I, as wide as III; V shorter than IV as long as VI. Antennomeres IV–VII nearly of same width, distal antennomeres slender, middle antennomeres as wide as apical.

**Figures 6–8.**
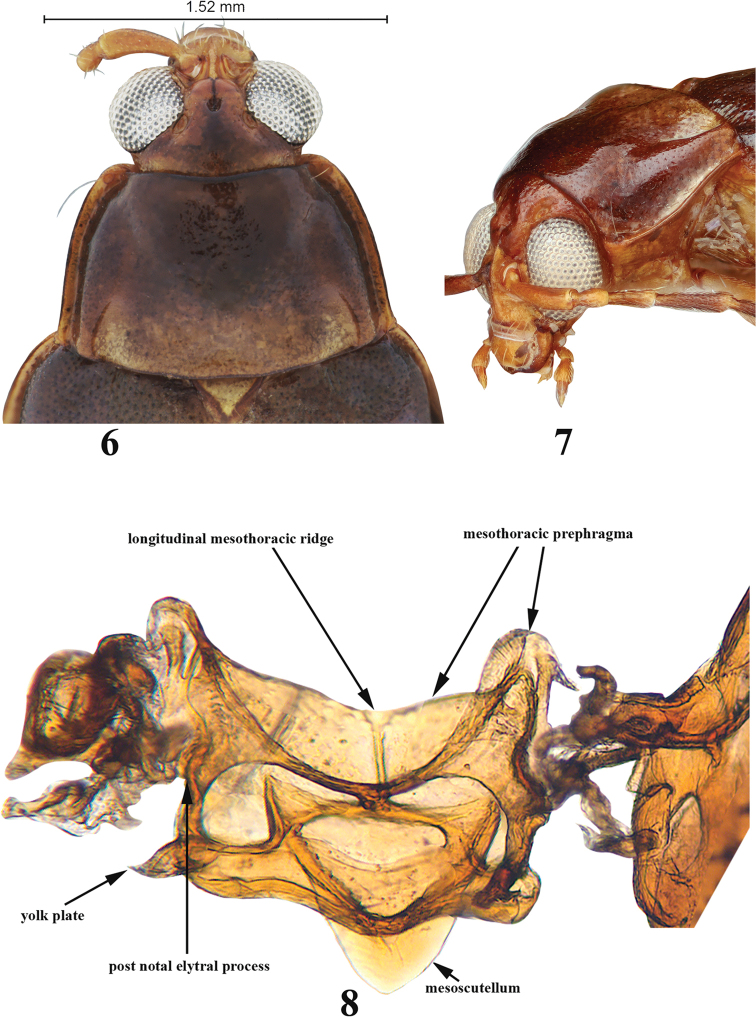
Adult *Blakealtica fusca*. **6** Pronotum **7** pronotum, three-quarter view **8** mesotergite.

***Prothorax*** (Fig. 6): Pronotal surface glabrous. Anterolateral callosity not expanding beyond lateral margin. Anterior setiferous pore situated close to anterior margin. Lateral margin of pronotum even, complete, nearly straight to gently convex, moderately explanate. Base of pronotum with two well developed longitudinal impressions, both near basal margin and further anteriorly. Pronotal basal margin evenly convex, antebasal transverse impression on pronotum shallow, present only near and limited by longitudinal impressions. Posterolateral callosity situated on lateral margin. Pronotal surface anteriorly uneven with two oblique impressions. Procoxal cavities open. Intercoxal prosternal process very narrow, procoxae almost touching, extends beyond procoxae, sides of process parallel; surface and posterior end convex; posterior end approximately as wide as in middle.

***Mesothorax*** (Figs 8, 10): Mesotergite 1.3 times wider than long. Longitudinal mesothoracic ridge about as long as mesoscutellum. Elytra at base wider than base of pronotum, punctures confused. Surface without hairs, or only small and indistinct hairs. Elytral sides nearly straight, parallel to each other. Scutellum present, relatively small. Humeral calli well developed, basal callus present as vague broad convexity behind scutellum. Transverse impression posteriad to humeral or basal callus absent. Lateral carina well developed, extends from humerus to basal three fourths of elytron. Epipleura gradually narrowing from base to apex, outwardly oblique, width less than that of profemur, basally wider than apically, reaches end of side of elytron, but not apex. Mesosternite approx. as wide as long, without elevated projection in middle, mesoventral process narrow, tip narrowly incised, mesocoxae nearly touching each other. Mesocoxal cavity open laterally.

**Figures 9, 10.**
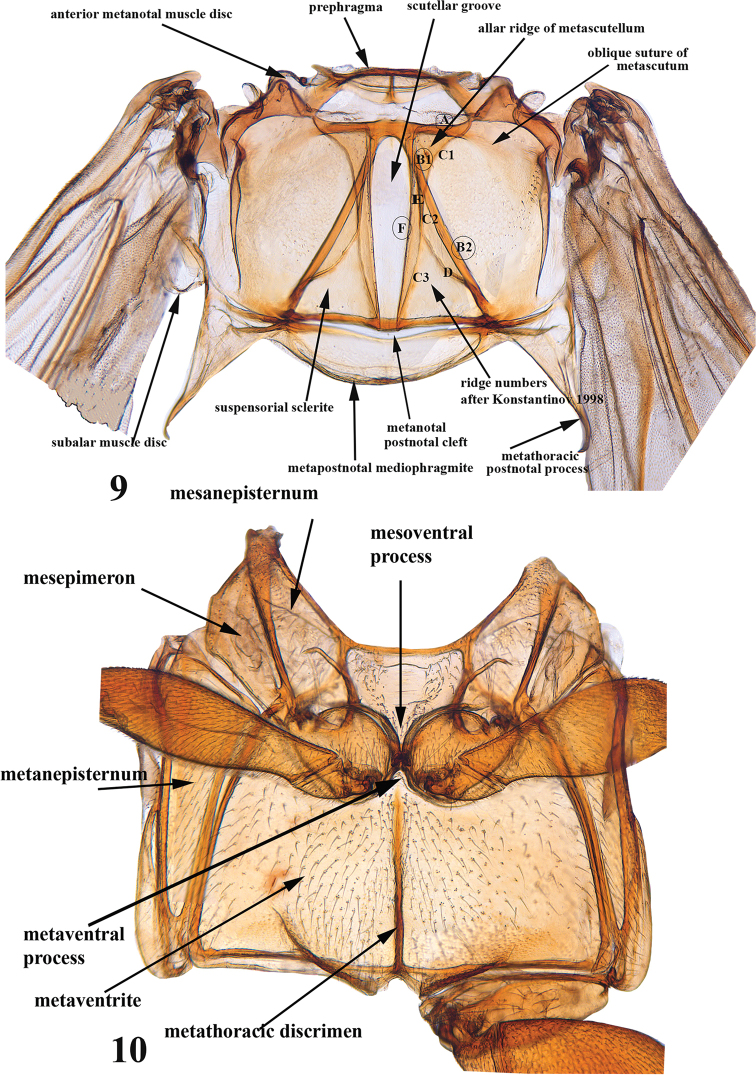
Adult *Blakealtica fusca*. **9** Metatergite **10** meso- and meta-sternites.

***Metathorax*** (Figs 9, 10, 20): Metatergite nearly as long as wide as measured in middle of metapostnotal mediophragmite. Prephragma well developed, highly sclerotized. Allar ridge of metascutellum slender. Scutellar groove and surrounding region with complete set of ridges. Metasternum slightly shorter than half its width; anteriorly projecting forward, but not covering mesosternum; apex narrow and acute. Metasternum without elevated projection in middle. Posterior end of metasternum flat. Metathoracic discrimen as long as entire length of metasternum. Metendosternite fully developed with arms at base much wider than stem near arms. Stem nearly as long as arm on its anterior side. Mid-tendons closer to each other than to ends of arms (Fig. 20).

***Abdomen*** (Figs 18, 19): Abdominal ventrites I and II not fused. Ventrites nearly equal in length. Ventrite I shorter than ventrites II and III together, ventrite V shorter than ventrites IV and III together. First abdominal ventrite between coxae without longitudinal ridges, apex of first abdominal sternite in female ogival.

***Legs*** (Figs 15, 16, 17): Pro- and meso-tibiae and femora not sexually dimorphic. Profemur generally flattened, but convex along ventral and dorsal surfaces. Pro- and meso-tibiae with longitudinal ridges, spurs absent. Posterior edge of metafemur in males as in that of females. Metafemoral spring present. Metatibia curved in dorsal view, straight in lateral view, more or less cylindrical in cross-section around middle, middle part of metatibia dorsally canaliculate. Dorsal side of metatibia with sharp edge laterally and mesally. Bristles of metatibial apex present laterally and mesally. Apex laterally blunt, does not form sharp spine. Metatarsomere I attached to apex of metatibia, single metatibial spur present, its length less than greatest width of metatibial apex, situated medially, spur simple, narrow, ending in one tooth. Protarsomere I of males wider and longer than in females. Protarsomere III slightly wider than protarsomere II. Metatarsomere III wider than long, roundish, incision of metatarsomere III longer than wide. Metatarsomere IV slender, similar to fourth pro- and meso-tarsomeres. Metatarsomere I in males long, cylindrical, length much less than half of metatibial length. Metatarsomere I and remaining tarsomeres produce an almost straight line. Claw appendiculate, not sexually dimorphic.

**Figures 11–16.**
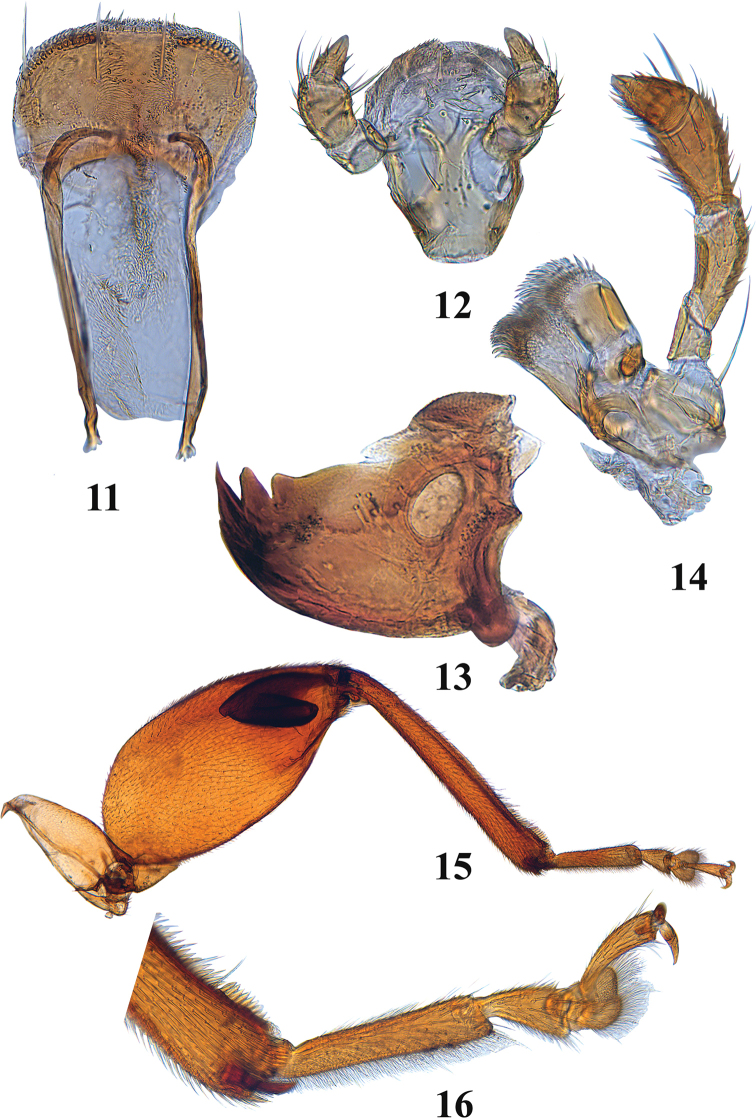
Adult *Blakealtica fusca*. **11** Labrum **12** labium **13** mandible **14** maxilla **15** hind leg **16** tip of hind tibia and hind tarsi.

***Genitalia*** (Figs 21–24): Spermathecal receptacle and pump with distinct border in between, receptacle longer than wide, spermathecal duct without coils, shorter than receptacle, attached to bottom of receptacle (Fig. 23). Tignum abruptly widened anteriorly (Fig. 24). Last visible tergite of female without longitudinal groove in middle (Fig. 19). Vaginal palpi not sclerotized and not visible. Median lobe of aedeagus sculptured, flattened with a knob at apex, dorsal opening very long, occupying nearly entire dorsal surface (Fig. 21).

**Figures 17–20.**
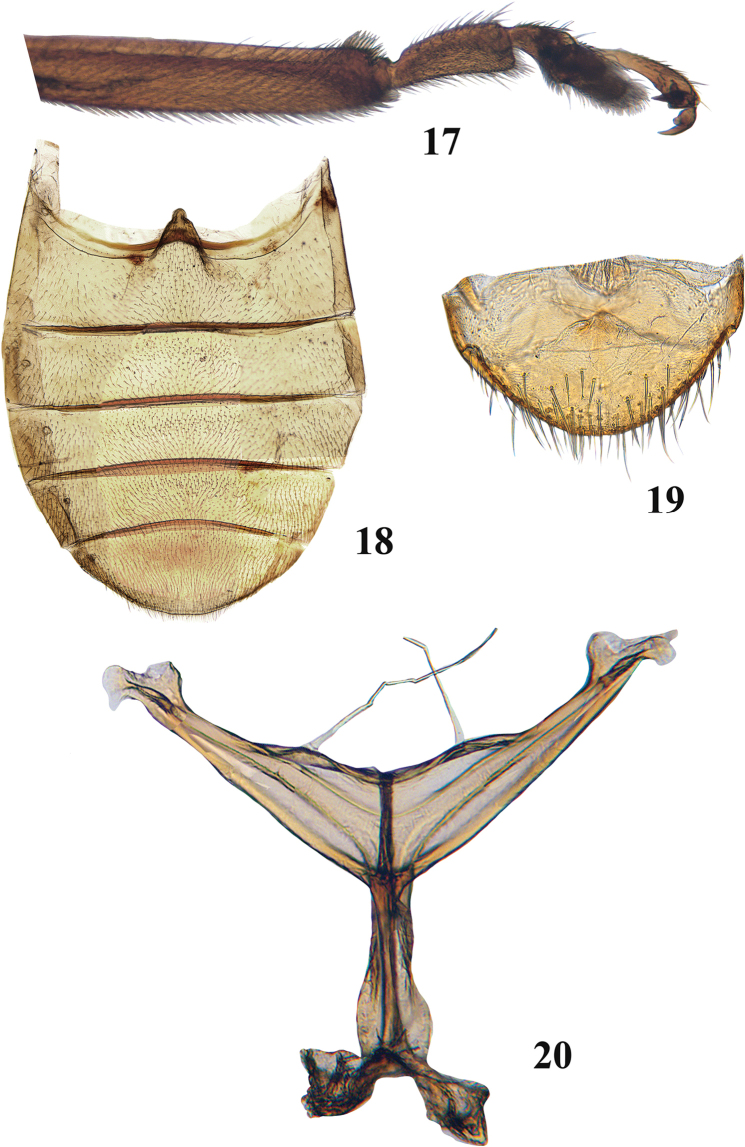
Adult *Blakealtica fusca*. **17** Tip of middle tibia and middle tarsi **18** female abdomen **19** apical abdominal tergite **20** metendosternite.

**Figures 21–24.**
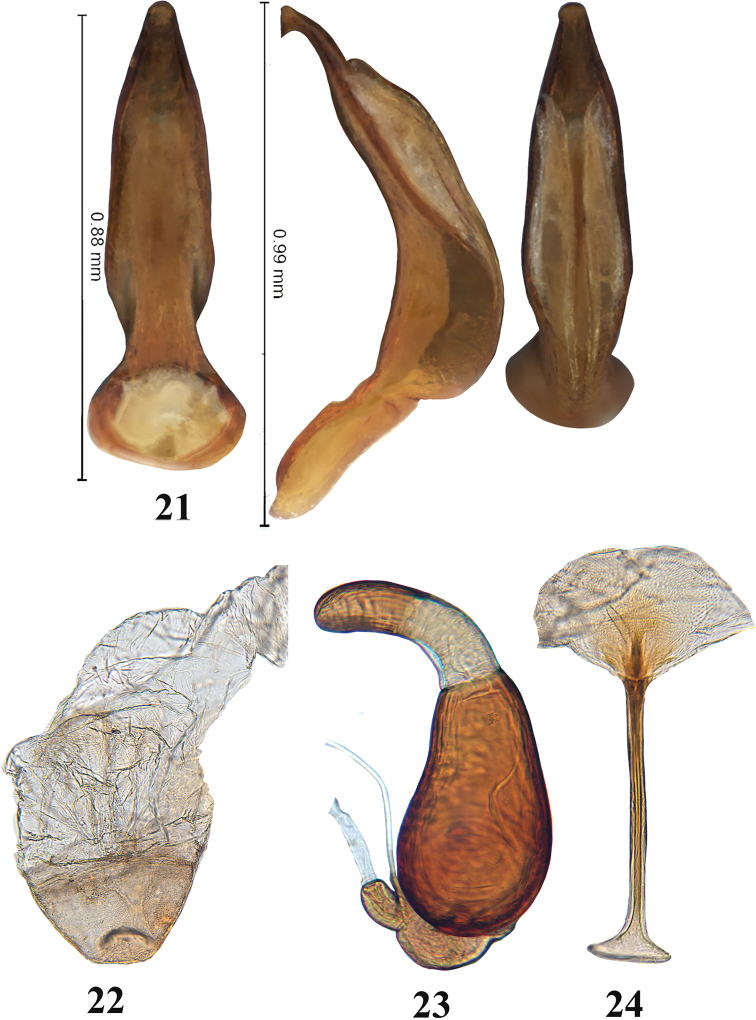
Adult *Blakealtica fusca*. **21** Median lobe of aedeagus (ventral, lateral, and dorsal views) **22** vagina **23** spermatheca **24** tignum.

#### Type species.

*Blakealtica fusca* sp. nov., by present designation.

#### Etymology.

We name this genus after Doris Holmes Blake (11 January 1892 – 3 December 1978) who, for almost 60 years of pioneering research of West Indian leaf beetles at the U.S. National Museum of Natural History, provided a foundation for leaf beetle taxonomy in the New World. She also built a significant portion of the leaf beetle collection at the Museum. The name is feminine.

#### Comparative diagnosis.

Because of the structure of sulci and ridges on the beetle’s head and the grooves on the beetle’s pronotum and general body shape, *Blakealtica* generally fits into the *Monomacra* group of genera as roughly defined by Bechyne & Springlova de Bechyne (1975). Therefore it is compared here to the following genera: *Monomacra* Chevrolat, 1836: 398 [type species *Haltica inermis* Klug, 1829: 9 by subsequent designation (Monros and Bechyne 1956:133)], *Disonycha* Chevrolat, 1836: 390 [type species *Crioceris collata* Fabricius, 1801: 463 by subsequent designation (Blake 1933: 1)], *Hemilactica* Blake, 1937 (type species *H. pulchella* Blake, 1937: 73 by original designation), *Parchicola* Bechyne & Bechyne, 1975: 63 (type species *Monomacra yena* Bechyne,1956: 1026, by original designation), *Pseudodisonycha* Blake, 1954: 248 (type species *Disonycha darlingtoni* Blake, 1938: 50, by original designation).

The studied representatives of all these genera do not have sclerotized vaginal palpi. Instead, the vagina has sclerotized walls and is therefore very noticeable on slides. A combination of these features may be unique for the aforementioned group of genera as we have not seen anywhere else in flea beetles. Very poorly sclerotized vaginal palpi were observed in a myrmecophilous flea beetle genus *Myrmeconycha* Konstantinov & Tishechkin, 2017 (type species *Myrmeconycha pheidole* Konstantinov & Tishechkin, 2017: 3). In addition, male genitalia and spermathecae of *Myrmeconycha* species are generally in the “style” of those of *Blakealtica*; however, the genera are very different in a variety of features (Konstantinov and Tishechkin 2017).

After studying the specimens of *Rosalactica maculicollis* (Jacoby, 1904), which is the type species of *Rosalactica* Bechyne & Bechyne 1977, it became clear that *R. maculicollis* is similar to *Blakealtica*, although we did not have an opportunity to study its female genitalia.

A key for identification of *Blakealtica* and the aforementioned genera is provided below. Since the genera within this group are not clearly or consistently differentiated, we tried to base the key on the type species of each genus; however, they were not always available (e.g., *Monomacra inermis*). Therefore, the key should be considered preliminary, although *Blakealtica* differentiates clearly and early on in the key.

### Blakealtica fusca sp. nov.

Taxon classificationAnimaliaColeopteraChrysomelidae

9726DFDE-DCC0-52C5-9C68-8F5DF89C4591

http://zoobank.org/CA115DA9-78E2-47AF-85A1-A70BE57DA270

Figures 1 , 2–5 , 6–8 , 9, 10 , 11–16 , 17–20 , 21–24

#### Description.

Body oblong, narrow, flat in lateral view, length 4.16–5.51 mm, width 1.72–2.27; thickness 1.18–1.51. General color dark yellowish to brown with light metallic blueish or pinkish tint. Elytra and pronotum same color. Vertex with punctures arranged in groups. Pronotal punctures large, and slightly deeper than those on vertex. Elytral punctures slightly larger and deeper than those on pronotum. Medial lobe of aedeagus in ventral view abruptly narrowing in basal third, widening towards middle and narrowing gradually to the apex, turning into narrow apex with a knob at it. In lateral view, median lobe bent at almost straight angle with knob pointing towards base. Large membranous slots on lateral view. Dorsal side of median lobe with large opening covered with two long lobes coming together along its length with a deep groove in between them. Vagina sclerotized apically in the shape of a dome, beyond which it is more membranous (Fig. 22). Spermathecal pump parallel sided, short, evenly rounded apically, slightly bent in lateral view. Spermathecal receptacle pear-shaped, sides symmetrical. Tignum widens abruptly anteriorly. Posteriorly, sclerotization merges with rest of sclerotized membrane.

#### Etymology.

The specific epithet refers to the generally brownish color of the beetles. It is a singular, feminine adjective in a nominative case.

#### Type material examined.

***Holotype*** female. 1) Dominican Republic, Zapoten, Villa Barroncolli, 16.XII 2014, 490 m, WP-520 18°17.886'N, 71°35.779'W Leg. N. Woodley; 2) ***Holotype****Blakealtica fusca* new species des. Konstantinov & Viswajyothi (USNM). ***Paratypes***: 1) Dominican Republic, Zapoten, night coll. 14.XII 2014, 408 m, WP-520 18°17.886'N, 71°35.779'W Leg. A. Konstantinov; 2) ***Paratype****Blakealtica fusca* new species des. Konstantinov & Viswajyothi (2 males USNM). 1) Dominican Republic, Independencia Prov., PN Sierra de Baoruco, (S of Puerto Escondido) 1215–400m 18°16.035'N, 71°32.684'W, 15.VII.04, leg. A. Konstantinov; 2) ***Paratype****Blakealtica fusca* new species des. Konstantinov & Viswajyothi (1 male USNM). 1) Dominican Rep.: Prov. Barahona, nr. Filipinas, Larimar mine, at light, 20–26-VI-1992 R. E. Woodruff & P. E. Skelley; 2) ***Paratype****Blakealtica fusca* new species des. Konstantinov & Viswajyothi (2 females, 1 male FSCA; 1 female MHND). The same label except for the dates, 26-VI–7-VII-1992 (4 females, 1 male FSCA).

### Key for identification of *Blakealtica* and related genera occurring in the Western Hemisphere

**Table:** 

1	Pronotum with a complex sculpture consisting of two longitudinal and two transverse ridges that connect to each other. Elytron with more than one longitudinal ridge. Dorsal surface covered with waxy substance.	***Myrmeconycha* Konstantinov & Tishechkin**
–	Pronotum without two longitudinal and two transverse ridges that connect to each other (Fig. 6). Elytron without longitudinal ridges, or with only one ridge. Dorsal surface not covered with waxy substance (Fig. 1)	**2**
2	Base of pronotum without transverse or longitudinal impressions	**3**
–	Base of pronotum with transverse or longitudinal impressions or both (Fig. 6)	**4**
3	Antennomeres beyond second cylindrical, antennomeres 4 and 5 not wider than apical antennomeres	***Disonycha* Chevrolat**
–	Antennomeres beyond second more or less flattened, antennomeres 4 and 5 wider than apical antennomeres	***Pseudodisonycha* Blake**
4	Head with mid-cranial suture present in lower part of vertex, represented by a short, relatively wide, deep depression (Figs 4, 5). Hind tibia dorsoventrally flattened with grove along its length (Fig. 15)	***Blakealtica* new genus**
–	Head without mid-cranial suture. Hind tibia more or less round in cross section, without grove along its length	**5**
5	Orbit extremely narrow. Frontal ridge long, extending lower than lower side and antennal sockets	***Rosalactica* Bechyne & Bechyne**
–	Orbit generally wide. Frontal ridge does not extend much lower than lower side of antennal sockets	**6**
6	Vertex covered with large closely-placed punctures. Elytra often with markings and longitudinal ridges	***Hemilactica* Blake**
–	Vertex covered with small distantly-placed punctures. Elytra often without markings, always without longitudinal ridges	**7**
7	Head with transfrontal sulcus absent or poorly impressed. Pronotum mostly with transverse impression. Elytra often with basal callus	***Monomacra* Chevrolat**
–	Head with transfrontal sulcus well impressed. Pronotum mostly without transverse impression. Elytra often without basal callus	***Parchicola* Bechyne & Bechyne**

## Supplementary Material

XML Treatment for Blakealtica

XML Treatment for Blakealtica fusca
